# Ursodesoxycholic acid alleviates liver fibrosis via proregeneration by activation of the ID1‐WNT2/HGF signaling pathway

**DOI:** 10.1002/ctm2.296

**Published:** 2021-01-24

**Authors:** Xi Dong, Yun Luo, Shan Lu, Han Ma, Wenchao Zhang, Yue Zhu, Guibo Sun, Xiaobo Sun

**Affiliations:** ^1^ Key Laboratory of Innovative Drug Discovery of Traditional Chinese Medicine (Natural Medicine) and Translational Medicine Institute of Medicinal Plant Development, Peking Union Medical College and Chinese Academy of Medical Sciences Beijing 100193 P. R. China; ^2^ Key Laboratory of New Drug Discovery Based on Classic Chinese Medicine Prescription Chinese Academy of Medical Sciences Beijing 100193 P. R. China; ^3^ Key Laboratory of Bioactive Substances and Resources Utilization of Chinese Herbal Medicine, Ministry of Education, Institute of Medicinal Plant Development Chinese Academy of Medical Sciences & Peking Union Medical College Beijing 100193 P. R. China; ^4^ Key Laboratory of Efficacy Evaluation of Chinese Medicine against Glycolipid Metabolic Disorders, State Administration of Traditional Chinese Medicine, Institute of Medicinal Plant Development Peking Union Medical College and Chinese Academy of Medical Sciences Beijing 100193 P. R. China; ^5^ School of Traditional Chinese Medicine Capital Medical University Beijing P. R. China; ^6^ College of Life Science and Technology Beijing University of Chemical Technology Beijing P. R. China

**Keywords:** ID1, HGF, WNT2, regeneration, ursodesoxycholic acid, liver fibrosis

## Abstract

**Background:**

The human liver possesses a remarkable capacity for self‐repair. However, liver fibrosis remains a serious medical concern, potentially progressing to end‐stage liver cirrhosis and even death. Liver fibrosis is characterized by excess accumulation of extracellular matrix in response to chronic injury. Liver regenerative ability, a strong indicator of liver health, is important in resisting fibrosis. In this study, we provide evidence that ursodesoxycholic acid (UDCA) can alleviate liver fibrosis by promoting liver regeneration via activation of the ID1‐WNT2/hepatocyte growth factor (HGF) pathway.

**Methods:**

Bile duct ligation (BDL) and partial hepatectomy (PH) mouse models were used to verify the effects of UDCA on liver fibrosis, regeneration, and the ID1‐WNT2/HGF pathway. An *Id1* knockdown mouse model was also used to assess the role of *Id1* in UDCA alleviation of liver fibrosis.

**Results:**

Our results demonstrate that UDCA can alleviate liver fibrosis in the BDL mice and promote liver regeneration via the ID1‐WNT2/HGF pathway in PH mice. In addition, *Id1* knockdown abolished the protection afforded by UDCA in BDL mice.

**Conclusions:**

We conclude that UDCA protects against liver fibrosis by proregeneration via activation of the ID1‐WNT2/HGF pathway.

## Graphical abstract

Critical points:

1. UDCA alleviates liver fibrosis and increased *Id1* mRNA expression in BDL mice model.

2. UDCA promotes liver regeneration and ID1‐WNT2/HGF signaling in PH mice model.

3. UDCA's antifibrosis and proregeneration effects are abolished in *Id1* knocking‐down BDL mice model.

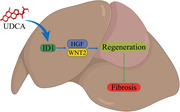

**Abbreviations**
AAV: adeno‐associated virus
ACLF: acute‐on‐chronic liver failure
ALP: alkaline phosphatase
BDL: bile duct ligation
CETSA: cellular thermal shift assays
CIFMS: CAMS Innovation Fund for Medical Sciences
ECM: extracellular matrix
H&E: hematoxylin and eosin
HGF: hepatocyte growth factor
HSC: hepatic stellate cell
IACUC: Institutional Animal Care and Use Committee
PH: partial hepatectomy

## 1 INTRODUCTION

The liver possesses a remarkable capacity for self‐repair following liver damage. The two different aspects of liver repair, regeneration and wound healing, function in harmony to manage any damage. While regeneration produces new liver cells, including hepatocytes, biliary epithelial cells, fenestrated endothelial cells, Kupffer cells, and cells of Ito,^1^ wound healing produces extracellular matrix (ECM) proteins in response to chronic injury. Chronic liver damage, which includes viral infections and alcohol and nonalcoholic steatohepatitis (NASH), can lead to fibrosis of the liver.^2^, ^3^, ^4^

Although fibrosis is a physiological repair process, fibrosis can be harmful or even progress to end‐stage cirrhosis when excessive or when aberrantly regulated during chronic injury.^4^, ^5^ Reduced liver regeneration is a feature of liver disease and is correlated with fibrogenesis.^6^ Resection of the liver by surgery, also known as partial hepatectomy (PH), triggers regeneration without fibrosis, because the regeneration of hepatic parenchyma outcompetes the growth of fibrous tissue.^7^ In a rat model, PH could accelerate the reversion of liver fibrosis through proregeneration.^8^ Likewise, parenchymal cell grafts can also stimulate liver regeneration and reduce fibrosis.^9^ Therefore, enhancing the regeneration of the liver can be considered a therapeutic strategy for liver fibrosis. Bile duct ligation (BDL) is a rodent model mimicking bile duct obstruction in humans (a clinically relevant event that can result in cholestatic injury). Because fibrogenesis and liver regeneration progress simultaneously in the BDL model,^6^ this model is an ideal tool for evaluating the protective role of liver regeneration against fibrosis.

The transcription factor, inhibitor of DNA binding 1 (ID1), is implicated in neural, epithelial, and hematopoietic stem cell proliferation and self‐renewal.^10^ In the liver, activation of *Id1* is essential for regeneration, inducing WNT2 and hepatocyte growth factor (HGF) expression after PH, and suppressing fibrosis caused by BDL.^2^, ^11^ In addition, the ID1‐WNT2/HGF pathway is involved in liver regeneration by promoting remote ischemic preconditioning after major PH.^12^

Ursodesoxycholic acid (UDCA), a hydrolytic product of tauroursodeoxycholic acid, is a major element obtained from the gall powder of bears. It is a traditional Chinese medicine used in clearing heat, in detoxification, and in improving vision. UDCA is the only drug approved by the US Food and Drug Administration for treating cholestatic liver diseases.^13^, ^14^ UDCA functions by eliminating hydroxyl radicals and inducing endogenous oxidation resistance, including elevating the expression of γ‐glutamylcysteine synthetase regulatory subunits, and elevating glutathione (GSH) synthesis.^14^ In addition, proregeneration is also considered to contribute to the antifibrosis effects of UDCA.^15^ To date, the role of *Id1* in UDCA function has not been elucidated.

In the present study, we investigate the connection between the antifibrosis and proregeneration effects of UDCA, and the important role *Id1* may play in these effects. We demonstrate that UDCA can protect the liver from fibrosis, and that this is accomplished by promoting hepatocyte regeneration via activation of the ID1‐WNT2/HGF pathway. Importantly, UDCA protection is abolished in the BDL mouse model following knockdown of *Id1*. Together, our study provides evidence that *Id1* plays a significant role in the antifibrosis effects of UDCA.

## 2 MATERIALS AND METHODS

### 2.1 Animal experiments, serum biochemical analysis, and histology

C57BL/6J wild‐type mice (male, 8‐ to 10‐week old) were purchased from Vital River Laboratories (Beijing, China) and raised in controlled light conditions (12 h light/12 h dark). Mice were provided free access to normal chow and water. The study protocol was approved by the Institutional Animal Care and Use Committee (IACUC) at the Chinese Academy of Medical Sciences and Peking Union Medical College, Beijing, China.

To determine the effects of UDCA on the BDL model, mice were grouped randomly and pretreated with either 15/30 mg/kg/day UDCA or solvent control by gavage for 5 days prior to BDL surgery.^14^ After the surgery, mice were treated with 15/30 mg/kg/day UDCA for another 3 days, fasted overnight, and then sacrificed. To analyze the effects of UDCA on the PH model, mice were grouped randomly and pretreated with 30 mg/kg/day UDCA or solvent control by gavage for 5 days prior to PH surgery. After surgery, mice were treated with 30 mg/kg/day UDCA for 2 days, fasted overnight, and then sacrificed.^14^ In both experiments, plasma and liver tissue were collected and stored at −80°C or fixed in 4% tissue fix solution (Coolaber, Beijing, China). Serum biochemical analysis was performed using an AU480 analyzer (Beckman Coulter, CA). For morphological analysis, the livers were processed for Hematoxylin and Eosin (H&E), Sirius red, and Masson's staining.^16^

### 2.2 Intrahepatic knockdown of *Id1*

Custom‐made adeno‐associated virus (AAV) harboring shRNA for mouse *Id1* (AAV*‐Id1*) and mouse nonsense control shRNA (AAV‐Ctrl) were obtained from Hanbio Biotechnology Co. Ltd. (Shanghai, China). AAV*‐Id1* shRNA and AAV‐Ctrl shRNA were injected intravenously into mice (1 × 10^8^ pfu) to knockdown *Id1* and as a control, respectively.

### 2.3 Immunohistochemical analysis

Immunohistochemical staining was performed using an Immunohistochemical Staining kit (ZSGB‐BIO, Beijing, China) according to the manufacturer's instructions. Liver tissue sections were stained with antibodies for Ki67 (Proteintech, Wuhan, China), collagen I (Abcam, London, UK), and F4/80 (BioLegend, CA). Quantification was performed using an Image‐pro plus (Meyer Instruments, TX).

### 2.4 Immunohistochemical analysis

Paraffin embedded sections were blocked with 5% goat serum and incubated with antibodies for Ki67 (Abcam) overnight at 4°C. The sections were then incubated with secondary antibody (Cell Signaling, MA) for 1 h at room temperature. Finally, images were obtained for analysis.

### 2.5 Terminal deoxynucleotidyl transferase‐mediated dUTP nick‐end labeling assay

The terminal deoxynucleotidyl transferase‐mediated dUTP nick‐end labeling (TUNEL) assay was performed according to the manufacturer's instructions provided with the in situ cell death detection kit (Roche, Basel, Switzerland). Briefly, liver tissue sections were permeabilized and incubated with TUNEL reaction mixture. After the addition of converter‐POD and substrate solution, liver tissue sections were analyzed using microscopy; quantification was performed using Image‐pro plus (Meyer Instruments, TX).

### 2.6 Quantitative real‐time polymerase chain reaction

Total RNA was isolated from frozen liver tissues using Trizol reagent according to the manufacturer's instructions (Invitrogen, CA). cDNA was synthesized from RNA using the PrimeScript RT Reagent kit (Takara, OSA, Japan). All samples were subsequently analyzed using a LightCycler 480 (Roche, Basel, Swiss).^14^

### 2.7 Cell cycle analysis

Fresh liver tissue was grounded and filtered using a 300 mesh to obtain a single‐cell suspension, which was subsequently fixed using 75% precooled alcohol and stored at 4°C overnight. The fixed suspension was stained using the cell cycle and apoptosis analysis kit (Beyotime, Beijing, China) according to the manufacturer's instructions. All samples were subsequently analyzed using flow cytometry (BD FACSCalibur, NJ).

### 2.8 Cellular thermal shift assay

The thermal shift assay was conducted as follows. Briefly, 100 μL aliquots of C57BL/6J wild‐type mouse liver lysate was mixed with 10 μM UDCA and incubated at different temperatures (42, 47, 52, 57, 62, or 67°C) for 3 min. The samples were subsequently centrifuged for 15 min at 12 000 rpm to separate the supernatants. The supernatants were then mixed with loading buffer and ID1 expression was analyzed using SDS‐PAGE.

### 2.9 Cell culture

HepG2 cells were purchased from Guan Dao Biotechnology (Shanghai, China), and cultured in DMEM (HyClone, MA) with 10% fetal bovine serum (Thermo Fisher Scientific, MA) at 37°C and 5% CO_2_.

### 2.10 Cell viability assay

HepG2 cells were cultured in 96‐well plate in condition described in 2.9. A total of 400μM to 3.13μM UDCA were incubated with HepG2 cells for 10 h. Then supernatant was discarded, and 10× diluted CCK‐8 solution (Solarbio, Beijing, China) was added to each well. After incubation for 2 h, OD values were obtained, and viabilities were calculated.

### 2.11 Molecular docking

The crystal structure of ID1 (PDB ID, 6MGN) was obtained from the Protein Data Bank, and the structure of UDCA (PubChem ID 31401) was obtained from the PubChem database. ID1 and UDCA structures were prepared using AutoDockTools 1.5.6 (Molecular Graphics Laboratory, CA). The docking parameters were set as previously described.^17^ The optimal docking result was visualized using PyMOL software (The PyMOL Molecular Graphics System, Version 2.0 Schrödinger, LLC).

### 2.12 Western blotting

Total liver protein was isolated using RIPA lysis buffer (CWBIO, Beijing, China) containing protease inhibitor cocktail (CWBIO). Equal amounts of protein were separated using 8% SDS‐PAGE gel electrophoresis in a Bio‐Rad Mini‐Protean system. The separated proteins were then transferred to nitrocellulose membrane (Millipore, MA) and incubated with specific primary antibodies (aSMA, ID1, HGF, WNT2, c‐MET, GSK‐3β, Proteintech, Wuhan, China; phosphor‐c‐MET, phosphor‐GSK‐3β, Abclonal, Wuhan, China). After washing and incubation with the secondary antibody, antibody complexes bound to specific liver proteins were visualized using the BIO‐RAD Gel Doc XR+ system (Bio‐Rad, CA).

### 2.13 Statistical analyses

Statistical Product and Service Solutions (IBM SPSS) software was used to test the normality and homogeneity of variance using the Kolmogorov–Smirnov and Levene's test. Data from the two groups were compared using a *t*‐test. Data from four groups were compared using one‐way ANOVA on GraphPad Prism (GraphPad Software Inc., San Diego, CA), using Dunnett's post‐hoc test to analyze the difference between groups. Data are presented as mean ± SD. Differences were considered significant when the *P* value was <.05.

## 3 RESULTS

### 3.1 UDCA protected BDL‐induced liver fibrosis

**FIGURE 1:**
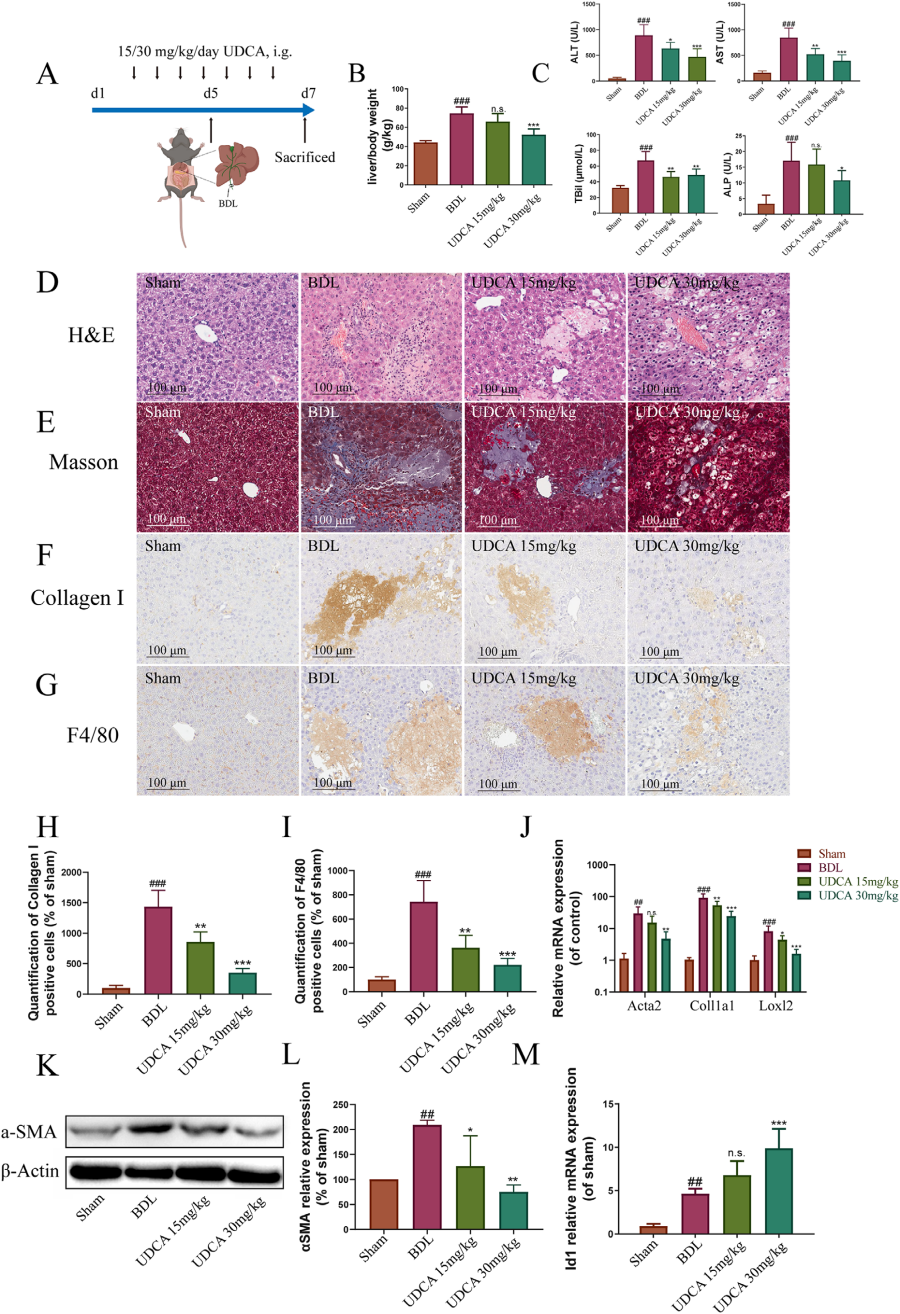
UDCA alleviated cholestatic liver fibrosis. (A) Experimental design demonstrating UDCA treatment of BDL mice. (B) UDCA improved liver to body weight ratio in BDL mice (n = 5). (C) UDCA ameliorated ALT, AST, TBil, and ALP levels in BDL mice (n = 5). (D) Hematoxylin & Eosin staining of the liver in BDL mice (with and without UDCA treatment). (E) Masson's staining of the liver in BDL mice (with and without UDCA treatment). (F) Reduction in collagen I deposition in BDL livers following UDCA treatment. (G) Reduction in F4/80 expression in BDL livers following UDCA treatment. (H) Quantification of collagen I positive cells in BDL livers (n = 5). (I) Quantification of F4/80 positive cells in BDL livers (n = 5). (J) Inhibition of the expression of hepatic stellate cell activating genes following UDCA treatment. (K) Inhibition of α‐SMA expression in BDL liver following UDCA treatment. (L) Statistical analysis of western blot (n = 5). (M) Increased transcription of *Id1* in BDL mice following UDCA treatment. All plots are presented as the mean ± SD. #*P *< .05, ##*P *< .01, ###*P *< .001, compared with the sham group; *P < .05, **P < .01, ***P < .001, compared to the BDL group

The experimental arrangement is illustrated in Figure 1A. The success of BDL surgery in a mouse model was evidenced by elevated liver to body weight ratios. The increase in liver to body weight ratio reflected an augmentation of liver weight owing to structural changes.^18^ In addition, observed elevations in alanine aminotransferase (ALT), aspartate aminotransferase (AST), total bilirubin (TBil), and alkaline phosphatase (ALP) levels provided further evidence of liver injury in the BDL mice (Figure 1C).

The liver to body weight ratio was significantly reduced following treatment with 15 mg/kg UDCA. The ratio was further reduced to a nonsignificant level (1.2‐fold of sham group) following treatment with 30 mg/kg UDCA. In addition, 30 mg/kg UDCA could decrease ALT, AST, TBil, and ALP levels, providing evidence of comprehensive protection against injury (Figure 1C). Hematoxylin‐eosin staining (H&E, Figure 1D) provided additional evidence of the improved status of the liver following UDCA treatment. Likewise, Masson's staining demonstrated the alleviation of fibrosis in the liver following treatment with UDCA (Figure 1D, E).

IHC staining of collagen I was also in line with those presented in previous studies, indicating reduced synthesis of collagen I (Figure 1F, H). Inflammation is indicative of cholestatic liver injury.^4^ Based on F4/80 staining, it was found that although macrophages were overly abundant in the liver with BDL, inflammatory cell recruitment was inhibited by UDCA treatment (Figure 1G, I). In agreement with the results of liver histology, hepatic stellate cell (HSC) activation genes, including collagen I (*Coll1a1*), alpha smooth muscle actin (*Acta2*), and lysyl oxidase‐like 2 (*Loxl2*), were markedly reduced by UDCA treatment (Figure 1J). In addition, α‐SMA expression, which was elevated in BDL mice, was also significantly decreased following UDCA treatment (Figure 1K, L). Because of its proregeneration effects, upregulation of the *Id1* gene may be beneficial for liver fibrosis.^2^, ^12^ To assess the effects of *Id1*, we first examined changes in *Id1* gene expression following UDCA treatment. As shown in Figure 1M, a significant increase in *Id1* gene transcription was observed in BDL mice. This was accompanied by an additional increase in *Id1* gene expression in UDCA treated BDL mice, suggesting a proregeneration role for UDCA in antifibrosis therapy.

### 3.2 UDCA promoted liver regeneration in the PH model

**FIGURE 2:**
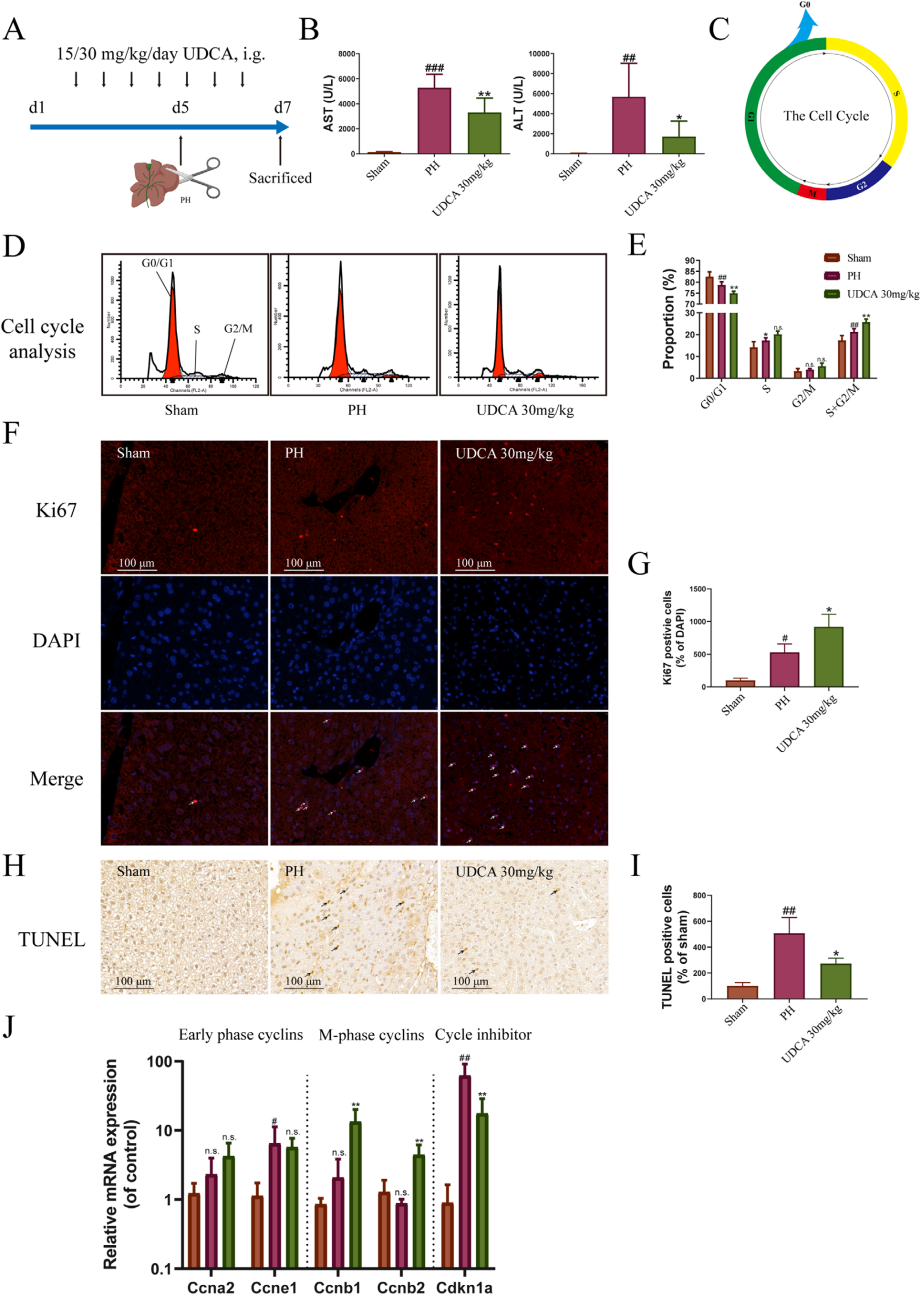
UDCA promotes liver regeneration in the partial hepatectomy model. (A) Experimental design for UDCA treatment of PH mice. (B) UDCA reduced serum aspartate transaminase and alanine aminotransferase levels in PH mice (n = 5). (C) Schematic diagram of the cell cycle. (D) Images of cell cycle assay using flow cytometry (n = 5). (E) Statistical analysis of cell cycle assay results, indicating that UDCA promotes cell cycle entry in PH liver (n = 5). (F) UDCA increased Ki67 expression in PH liver (n = 5). (G) Quantification of Ki67 positive cells in liver (n = 5). (H) TUNEL assay demonstrating that UDCA treatment alleviates apoptosis in PH livers. (I) Quantification of TUNEL‐positive cells in liver. (J) Effects of UDCA treatment on cell cycle cyclin genes and cell cycle inhibitor p21 (n = 5). All plots are presented as the mean ± SD. #*P *< .05, ##*P *< .01, ###*P *< .001, compared with the sham group; *P < .05, **P < .01, ***P < .001, compared with the PH group

Considering the important role of regeneration in the treatment of liver fibrosis, and upregulation of the *Id1* gene in BDL mice after UDCA treatment, we examined the impact of UDCA treatment on liver regeneration in the PH model. The experimental arrangement is illustrated in Figure 2A. Based on the comprehensive protection afforded by a 30 mg/kg dosage of UDCA in the BDL model, PH mice were treated accordingly with 30 mg/kg UDCA. As shown in Figure 2B, aspartate transaminase (AST) and alanine aminotransferase (ALT) levels increased following hepatectomy, indicating severe liver injury. UDCA treatment alleviated liver injury, decreasing AST and ALT levels (Figure 2B). Thus, UDCA has a protective role in the PH model.

A cell fate switch determines the choice to cycle (proliferate) into the G1 phase (the beginning of cell cycle, Figure 2C) followed by the S, G2, and M phases, or into the quiescent state (G0).^19^ Owing to the limitations of flow cytometry, it was not possible to distinguish cells in G0 from those in G1 (or G2 from M), as these states possess the same amount of DNA. In contrast, the S and G2/M phases can be identified as mitotic cells, as cells in this state have more DNA than cells in the G0 or G1 phases.^19^ As shown in Figure 2D, E, the proportion of cells in G0/G1 phases decreased following hepatectomy, and UDCA treatment decreased the proportion further. In contrast, the proportion of S phase liver cells was elevated following a hepatectomy. Moreover, UDCA treatment slightly increased (but not significantly) the proportion of cells in the S phase (compared to that in the PH group). Although UDCA treatment slightly increased the proportion of cells in G2/M phases, no difference was observed among the three groups. However, UDCA treatment considerably increased the proportion of cells in S + G2/M phases (compared to that in the PH group).

Although our cell cycle analysis demonstrated no obvious differences in the proportion of cells in the S or G2/M phases between the PH and UDCA groups, the S + G2/M phases proportion of cells (representing total proliferative hepatocytes) were significantly increased following UDCA treatment (compared to the PH group). The results of our immunohistochemistry analysis using the proliferative marker, Ki67, confirmed these results. Thus, UDCA treatment significantly increased Ki67 expression in BDL mice (Figure 2F, G), consistent with a boost in the number of proliferative hepatocytes.^12^ Together, these results suggested that UDCA may play a positive role in promoting cell cycle entry.

Apoptosis generally plays an important role in regeneration.^20^ To understand the specific role of apoptosis in liver regeneration, we evaluated the extent of apoptosis in liver cells using the terminal deoxynucleotidyl transferase‐mediated dUTP nick‐end labeling (TUNEL) assay. As shown in Figure 2H, I, no obvious apoptosis was observed in liver cells from the sham group. However, apoptosis was amplified in the PH group following hepatectomy, which was consistent with the blood biochemistry results. In contrast, apoptosis was reduced in the UDCA treatment group, which was consistent with an improvement after liver injury. The demonstration of apoptosis following hepatectomy also implied a driving force to the regeneration.

Analysis of cell cycle gene expression provided additional evidence of a proregeneration role for UDCA. As shown in Figure 2J, hepatectomy enhanced the transcription of *Ccne1*, a member of the early phase cyclins responsible for the G1/S transition.^21^ In contrast, hepatectomy had no apparent effect on M‐phase cyclins (*Ccnb1, Ccnb2*). In addition, cycle inhibitor p21 (*Cdkn1a*) was markedly elevated after hepatectomy. This increase in *Cdkn1a* expression may be responsible for the observed proliferation of hepatocytes and insufficient cell cyclin transcription. UDCA treatment exerted a similar effect (to hepatectomy) on early phase cyclins, an enhanced effect on M‐phase cyclins, and a considerable decrease in *Cdkn1a* expression.

After confirmation of the antifibrosis and proregeneration effects of UDCA in BDL and PH models, the effects of UDCA treatment on healthy animals were examined. In particular, we investigated whether UDCA influenced ID1, WNT2, and HGF expression independent of surgery. As shown in Supporting information Figure S1, a slight but significant increase in *Id1* transcription was observed after treatment with UDCA. In contrast, *Wnt2* and *Hgf* expression remained unchanged. Furthermore, although the expression of all three proteins was elevated, the observed increases were not significant.

### 3.3 UDCA enhanced CXCR7‐ID1‐WNT2/HGF signaling in the PH model

**FIGURE 3:**
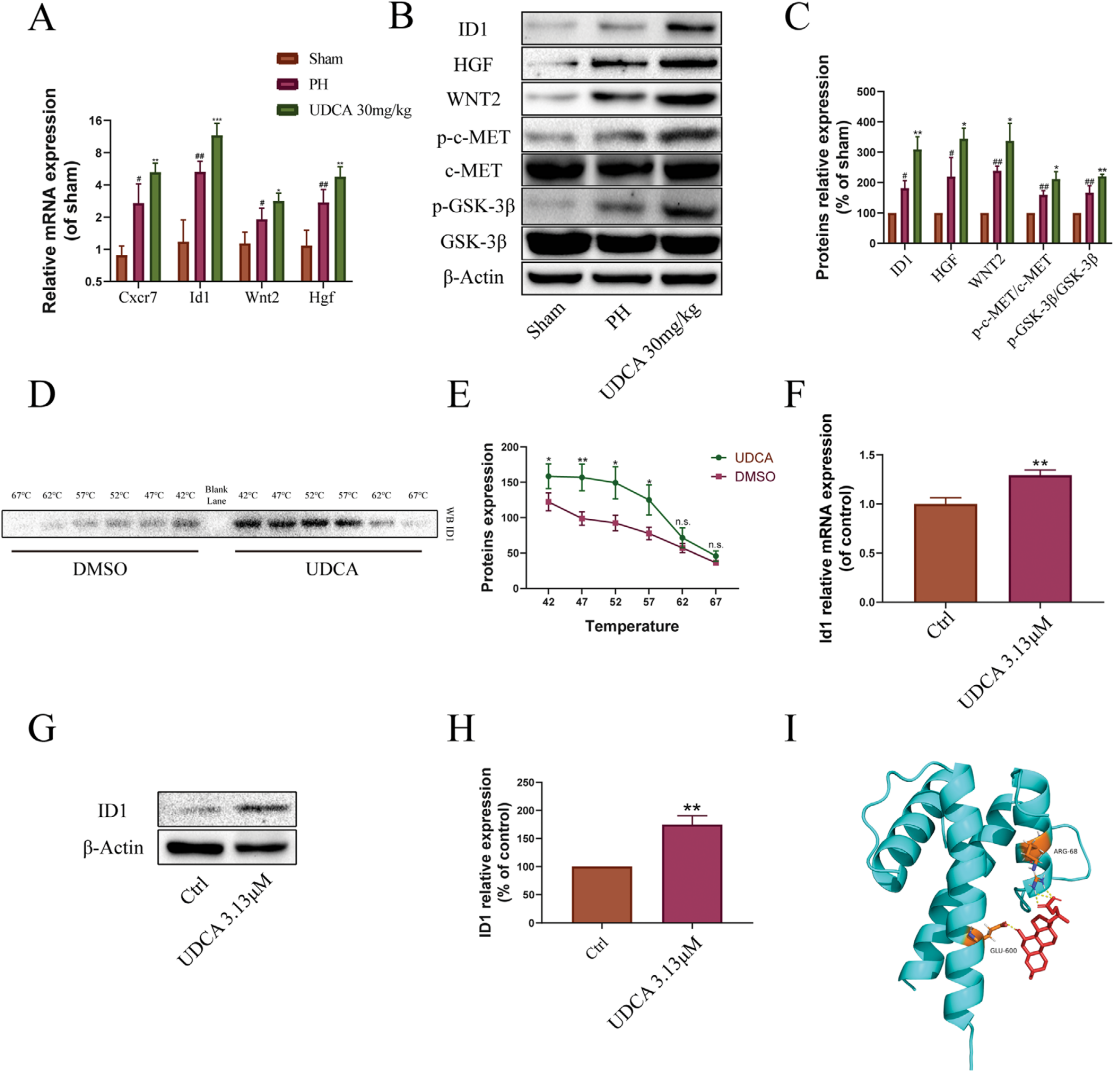
UDCA enhanced ID1‐WNT2/HGF signaling and ID1 thermal stabilization. (A) UDCA enhanced the expression of genes in the CXCR7‐ID1‐WNT2/HGF signaling pathway (n = 5). (B) UDCA enhanced the expression of proteins in the CXCR7‐ID1‐WNT2/HGF signaling pathway and the phosphorylation of c‐MET and GSK‐3β (n = 5). (C) Statistical analysis of western blotting results (n = 5). (D) UDCA treatment enhanced the thermal stabilization of ID1 at different temperatures (CETSA, n = 5). (E) Statistical analysis of western blotting results for CETSA. (F) UDCA treatment elevated the transcription of *Id1* gene in HepG2 cells (n = 3). (G) UDCA treatment elevated the expression of ID1 protein in HepG2 cells. (H) Statistical analysis of western blotting results (n = 3). (I) Predicted UDCA‐ID1 interaction. Key interface residues (ARG‐68 and GLU‐600) in ID1 are shown as sticks and marked in dark yellow. The yellow dotted lines indicate polar contacts between UDCA and ID1 predicted by the software. All plots are presented as the mean ± SD. #*P *< .05, ##*P *< .01, ###*P *< .001, compared with the sham group; *P < .05, **P < .01, ***P < .001, compared with the PH, DMSO, or control groups, n.s., nonsignificant

CXCR7‐ID1‐WNT2/HGF signaling is involved in hepatic regeneration in the PH model, and also plays a positive role in alleviating liver fibrosis.^2^, ^22^ Therefore, we examined whether the proregeneration effects of UDCA were related to CXCR7‐ID1‐WNT2/HGF signaling in the PH model.^2^, ^12^, ^22^ As shown in Figure 3A, UDCA treatment significantly increased *Id1* expression, and *Hgf* and *Wnt2* expression, two downstream targets of *Id1*. Consistent with these observations, western blotting confirmed the elevated expression of ID1, HGF, and WNT2 proteins (Figure 3B, C).

Because we observed an increase in WNT2 and HGF, phospho‐GSK‐3β and phospho‐c‐MET levels were also determined. GSK‐3β hinders the activation of WNT2 signaling by phosphorylation and subsequent degradation of β‐catenin. Phosphorylation of GSK‐3β attenuates enzymatic activity and stability, resulting in an accumulation of β‐catenin, and activation of WNT2 signaling.^23^, ^24^ c‐MET is a tyrosine kinase receptor for HGF. HGF binding to c‐MET results in the autophosphorylation of c‐MET.^25^, ^26^, ^27^ As seen in Figure 3B, C, phospho‐GSK‐3β/GSK‐3β and phospho‐c‐MET/c‐MET were all increased, confirming WNT2 activation and HGF signaling. Therefore, our data provide evidence of UDCA‐activated CXCR7‐ID1‐WNT2/HGF signaling in the liver leading to the promotion of liver regeneration.

### 3.4 UDCA improved thermal stabilization of ID1

ID1 plays a role in the effects of UDCA in both the BDL and PH mouse models. To understand this role further, we investigated whether there was direct interaction between ID1 and UDCA. Cellular thermal shift assays (CETSA) were used to evaluate the interaction between UDCA and ID1, based on the principle that drug binding can increase the thermal stability of target proteins.^28^ As shown in Figure 3D, E, UDCA enhanced the thermal stability of ID1 at 42, 47, 52, and 57°C (no effect was observed with DMSO control). The CETSA result provides evidence of a direct interaction between UDCA and ID1. Then, we sought to determine the impact of UDCA on ID1 at the cellular level though the upregulation of ID1 by UDCA was verified in the PH model. The viability of HepG2 cells was tested after treatment with different doses of UDCA. As was shown in Supporting information Figure S2, high doses (400 and 200 μM) of UDCA inhibited the viability of HepG2 cells, while low doses (6.25 and 3.13 μM) promoted the proliferation of HepG2 cells. Next, we examined UDCA's effects on ID1 at gene and protein levels. First, we evaluated the effects of 3.13 μM UDCA on *Id1* transcription because this dose exerted dramatic proliferation promotion effect in the viability assay. The results indicated 3.13μM UDCA increased *Id1* transcription significantly (Figure 3F). Then, the expression of ID1 was checked after treatment with 3.13μM UDCA. As was shown in Figure 3G, H, ID1 expression was elevated after treatment with UDCA. Next, we used AutoDockTools software to construct a model of the UDCA‐ID1 complex (Figure 3I). The optimized docking model predicted contacts between UDCA and ARG‐68 and GLU‐600 of ID1.

### 3.5 *Id1* knockdown abolished UDCA protection from BDL‐induced liver fibrosis

**FIGURE 4:**
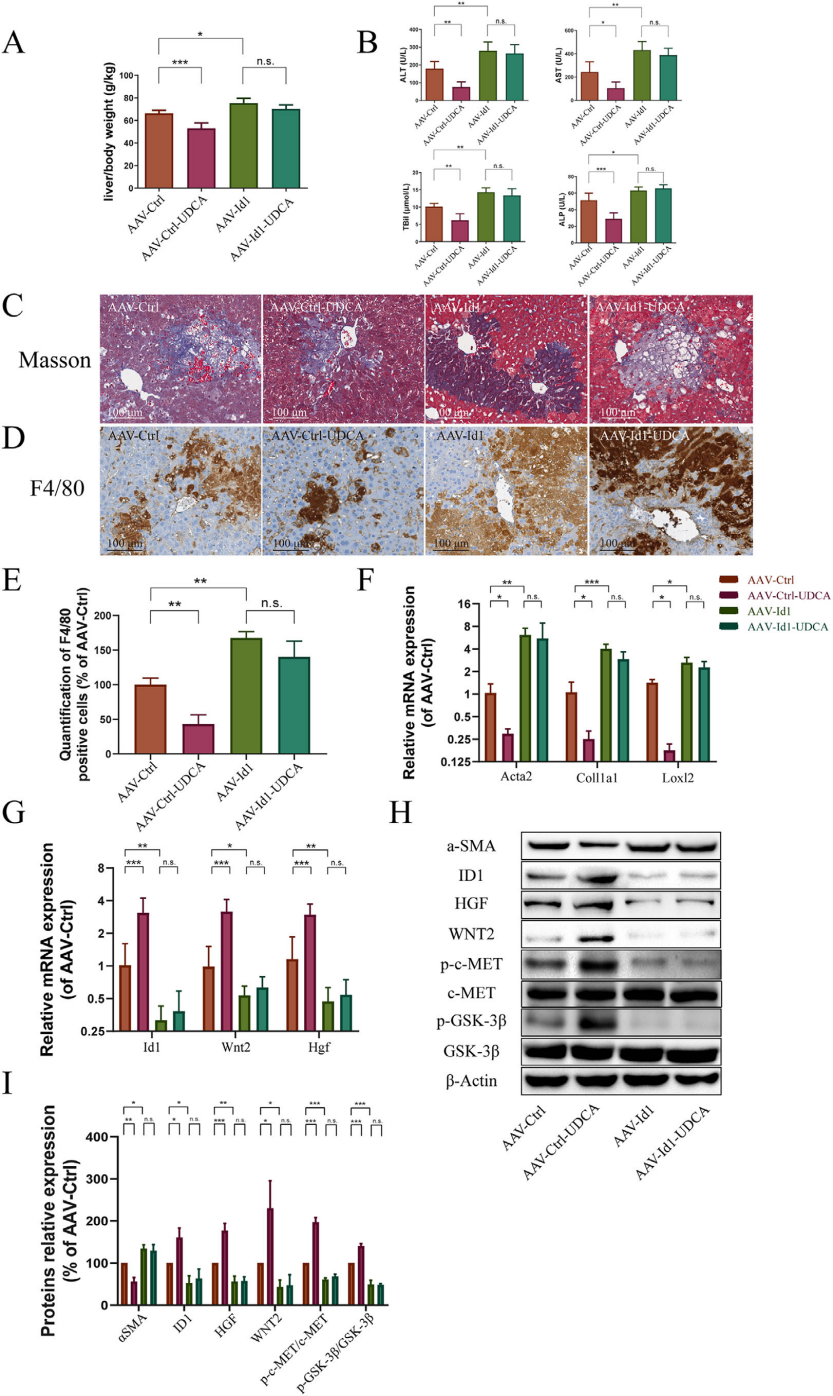
Knockdown of *Id1* abolished UDCA protection in BDL mice. (A) *Id1* knockdown reversed the effects of UDCA on the liver to body weight ratio in BDL mice (n = 5). (B) *Id1* knockdown abolished UDCA‐induced improvements in serum ALT, AST, TBil, and ALP levels (n = 5). (C) Masson's staining demonstrated increased fibrosis in *Id1* knockdown livers following UDCA treatment compared to control mice treated with UDCA (n = 5). (D) F4/80 immunohistochemical staining demonstrated increased inflammation in *Id1* knockdown livers following UDCA treatment compared to control mice treated with UDCA (n = 5). (E) Quantification of F4/80 positive cells in liver (n = 5). (F) *Id1* knockdown abolished the effects of UDCA on HSC activating genes (n = 5). (G) *Id1* knockdown abolished the effects of UDCA on ID1‐WNT2/HGF signaling pathway gene transcription (n = 5). (H) *Id1* knockdown abolished the effects of UDCA on α‐SMA expression, ID1‐WNT2/HGF signaling pathway protein expression, and phosphorylation of c‐MET and GSK‐3β (n = 5). (I) Statistical analysis of western blotting results (n = 5). All plots are presented as the mean ± SD. *P < .05, **P < .01, ***P < .001, compared with the corresponding group

To confirm the role of *Id1* in UDCA protection from liver fibrosis, mice were injected with AAV*‐Id1* shRNA to knockdown *Id1* (AAV*‐Id1*) in vivo. As a control, mice were injected with empty AAV control shRNA (AAV‐Ctrl). The *Id1* knockdown procedure was first verified using liver tissue (Supporting information Figure S3). Next, the antifibrosis effects of UDCA were evaluated in gene‐modified mice following BDL surgery with or without treatment with 30 mg/kg UDCA. In mice treated with AAV control shRNA, decreases in the liver to body weight ratio and in the ALT, AST, TBil, and ALP levels were observed (Figure 4A, B). However, in mice treated with AAV*‐Id1* shRNA, no changes were observed (Figure 4A, B). In addition, while fibrosis and inflammation were both alleviated by UDCA treatment of AAV‐Ctrl shRNA injected mice (according to Masson's and F4/80 staining), UDCA protection was reversed in the *Id1* knockdown (Figure 4C to E). Furthermore, although UDCA reduced HSC activation gene expression (Figure 4F) and α‐SMA expression (Figure 4H, I) in control mice, these effects were not observed in AAV*‐Id1* shRNA‐injected mice. ID1‐WNT2/HGF signaling was also impaired in *Id1* knockdown mice, as evidenced by reduced levels of component mRNA and protein (Figure 4G to I). The observed changes in phospho‐GSK‐3β/GSK‐3β and phospho‐c‐MET/c‐MET were in line with a reduction in ID1‐WNT2/HGF signaling, further confirming the role of ID1‐WNT2/HGF signaling in UDCA action (Figure 4H, I). Together, the above data demonstrate that *Id1* knockdown abolished UDCA protection in the BDL model, suggesting that UDCA alleviated liver fibrosis via ID1‐WNT2/HGF signaling.

### 3.6 *Id1* knockdown abolished UDCA‐induced proregeneration in the BDL model

**FIGURE 5:**
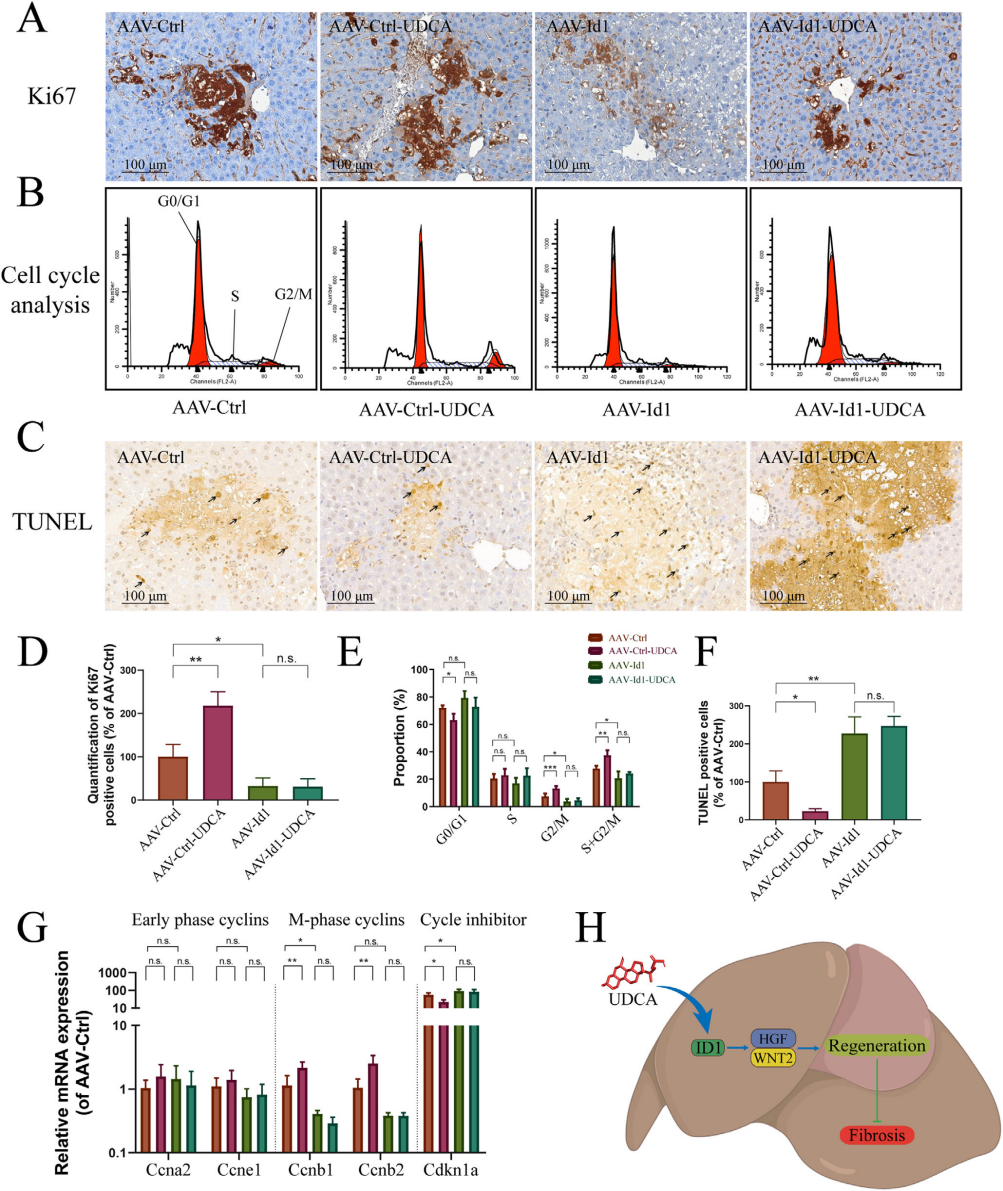
The proregeneration effect of UDCA was reversed by *Id1* knockdown. (A) UDCA promotion of Ki67 expression was abrogated by *Id1* knockdown (n = 5). (B) Cell cycle assay of the effects of UDCA on control or *Id1* knockdown livers. (C) TUNEL assay demonstrates that UDCA rescue of apoptosis was abrogated by *Id1* knockdown (n = 5). (D) Quantification of Ki67 positive cells in control or *Id1* knockdown livers (n = 5). (E) Statistical analysis of cell cycle assay, indicating *Id1* knockdown abrogated UDCA promotion of cell cycle entry (n = 5). (F) Quantification of apoptosis in liver cells using a TUNEL assay. (G) *Id1* knockdown abrogated the effects of UDCA on cell cycle cyclin gene expression and cell cycle inhibitor p21 expression (n = 5). (H) Schematic diagram of the proposed mechanism of UDCA action. All plots are presented as the mean ± SD. *P < .05, **P < .01, ***P < .001, compared with the corresponding group

Next, we evaluated liver regeneration in BDL mice injected with AAV*‐Id1* shRNA or AAV control shRNA. As shown in Figure 5A, D, UDCA elevated Ki67 expression in AAV‐Ctrl shRNA injected mouse liver, but not in AAV*‐Id1* shRNA injected mouse liver. Cell cycle assays confirmed the key role played by *Id1* in UDCA function. While UDCA treatment decreased the proportion of cells in G0/G1 phases in the AAV‐Ctrl‐UDCA group, no effect was observed in the AAV*‐Id1*‐UDCA group (compared to the AAV*‐Id1* group, Figure 5B, E). No difference in the proportion of S phase cells was observed across all groups. The proportion of cells in G2/M phases was significantly elevated by UDCA in the AAV‐Ctrl‐UDCA group, but not in the AAV*‐Id1*‐UDCA group (compared to the AAV*‐Id1* group). An analysis of the proportion of cells in the S + G2/M phases depicted a similar mode of action for UDCA in proregeneration.

Next, we detected apoptosis in the livers of the different groups. Apoptosis was observed in all four groups. While UDCA alleviated apoptosis in AAV control shRNA injected mice, apoptosis was not alleviated by UDCA in AAV*‐Id1* shRNA injected mice (Figure 5C, F). This observation was in line with the levels of HGF and WNT2 in *Id1* knockdown mice, implying that protection via ID1‐WNT2/HGF signaling was diminished.

Analysis of cell cycle cyclins provided additional support for the suggestion that ID1 participated in the proregeneration effects of UDCA. As shown in Figure 5G, no difference in early phase cyclins (*Ccna2* and *Ccne1*) was observed across groups. However, although M‐phase cyclin (*Ccnb1* and *Ccnb2*) levels were elevated by UDCA in the AAV‐Ctrl‐UDCA group, no difference between AAV*‐Id1* and AAV*‐Id1*‐UDCA groups was observed. In contrast, cell cycle inhibitor P21 (*Cdkn1a*) was significantly decreased by UDCA treatment in the AAV‐Ctrl‐UDCA group. Again, cell cycle inhibitor P21 (*Cdkn1a*) levels remained unchanged in both *Id1* knockdown groups. Together, our data indicate that interfering *Id1* hindered the proregeneration and antifibrosis effects of UDCA, suggesting that UDCA might function through proregeneration via the ID1‐WNT2/HGF signaling pathway (Figure 5H).

## 4 DISCUSSION

UDCA is used in the treatment of cholestatic liver diseases, and functions by eliminating hydroxyl radicals, inducing endogenous oxidation resistance, and inhibiting intestinal absorption of bile acids.^14^, ^29^ As the only drug approved for the treatment of primary biliary cholangitis, UDCA has been shown to ameliorate serum hepatic biochemistries, postpone histological progression, and delay the development of esophageal varices.^13^, ^29^ Survival in primary biliary cholangitis patients who responded to UDCA was comparable to that of healthy people.^30^, ^31^ Indeed, a meta‐analysis comprising 4845 patients in long‐term cohort studies uncovered an overall transplant‐free survival of 88% at 5 years, 77% at 10 years, and 63% at 15 years.^32^ Unfortunately, the molecular mechanism of UDCA on primary biliary cholangitis is still obscure.

Liver fibrosis is characterized by ECM protein deposition and is associated with HSC activation and inflammatory cell infiltration.^33^ The most abundant protein in the ECM protein deposition is collagen I.^34^ Using a BDL mouse model, we demonstrated that UDCA could decrease transcription of *coll1a1* and other HSC activation genes. Collagen I expression was also reduced after UDCA treatment. Consistent with these results, fibrosis, as indicated by Masson's and Sirius red staining, was also improved after UDCA treatment. These results confirm the antifibrosis effects of UDCA. α‐SMA is an actin isoform and a particular marker of smooth muscle cell differentiation.^35^ Hence, α‐SMA expression can be used to investigate activated HSCs with a myofibroblastic phenotype.^36^, ^37^, ^38^ Our study also demonstrated inhibition of α‐SMA expression by UDCA.

Fibrosis is preceded by inflammation.^4^ Inflammation is essential to eliminate cell debris and stimulate the aggregation of wound‐healing cells in the liver. Nevertheless, extreme inflammation can impair the viability of hepatocytes and accelerate the growth of progenitors and myofibroblasts, laying the basis for carcinogenesis and progressive fibrosis.^39^ Our study showed that UDCA could decrease macrophage infiltration, as evidenced by downregulated F4/80 expression, which is a specific macrophage marker,^40^ in the liver. The role of macrophages, an innate inflammatory cell population in the immune system, in liver fibrosis has been studied extensively. Macrophages are believed to accelerate the process of liver fibrosis. In rats, hepatic macrophage exhaustion resulted in reduced myofibroblast activation and fibrosis after thioacetamide injury.^41^ In another study involving carbon tetrachloride (CCl_4_) induced hepatic injury, macrophage depletion was found to decrease myofibroblast numbers and to attenuate liver fibrosis, suggesting the profibrotic role of macrophages in this context.^42^ The observation that UDCA alleviated inflammation therefore suggested protective effect of UDCA on liver fibrosis.

Because liver regeneration proceeds without fibrosis after PH, liver regeneration is considered to be a therapeutic strategy in liver fibrosis.^2^, ^6^, ^9^ After injury, tissue regeneration was triggered so that damaged tissue could be replaced, a process called “compensatory proliferation.” During this process, mitogenic signals were first generated by apoptotic cells, and then compensatory proliferation occurred. Thereafter, healthy cells were produced and injured tissue was repaired.^20^ Here, we showed apoptotic liver cells in animals undergoing PH surgeries, which suggested a driving force of liver regeneration. Cell proliferation is also a key event for tissue regeneration, which requires cell cycle entry. Research also confirms cell cycle regulation as part of the regenerative mechanism.^43^ In this study, we demonstrated a positive role for UDCA in liver regeneration using a PH mouse model. UDCA treatment promotes liver cells to enter the cell cycle, and decreases apoptosis. There seemed to be a conflict between decreased apoptosis and enhanced cell division. In fact, HGF and WNT signaling were shown to possess antiapoptosis and proregeneration effects in numerous studies,^44^, ^45^, ^46^ and they were all elevated after treatment with UDCA in the liver.

The cyclin‐dependent kinase (CDK) inhibitor p21 protein (encoded by *Cdkn1a*) is a negative regulator of cell cycle progress. P21 binds to CDK‐cyclin complexes and inactivates it, which inhibits the phosphorylation of downstream targets of CDK‐cyclin thereby leading to cell cycle arrest.^47^ In our research, *Cdkn1a* was dramatically upregulated by more than 60 folds in PH model, and by only about 17 folds in UDCA group. This might also account for the remarkable proliferation ability of liver in the UDCA group. Furthermore, overexpression of p21 leaded to cell cycle arrest.^48^, ^49^ Growth inhibition and cell cycle arrest of head and neck squamouscarcinoma cells by antineoplastic drug could be reduced if cells are transfected with p21 antisense constructs.^50^ Together, our study indicated UDCA promoted liver cells proliferation and regeneration. So the possibility that UDCA alleviated liver fibrosis through proregeneration could not be excluded.

Literature had demonstrated that ID1 was a transcriptional regulator and had no DNA binding domain, but it could form heterodimers with basic helix‐loop‐helix (bHLH) transcription factors, which resulted in DNA binding suppression.^60^ HGF and WNT2 are downstream targets of ID1. However, there is no detailed explanation of the exact mechanism of how ID1 regulates WNT2 or HGF to date. Ding and colleagues found expressions of WNT2 and HGF were critically diminished in *Id1* ^−/−^ liver cells. Furthermore, *Id1* ^−/−^ liver cells transduced with HGF and WNT2 could restore the regeneration of mass and cell expansion in the *Id1* ^−/−^ liver.^22^ And *Id1* promoted WNT2 expression, which accelerated cell cycle progression by enhancing G1 to S transition.^11^ The WNT2 and HGF were also verified as targets of ID1 in resection‐induced liver failure in the mouse.^12^ We also demonstrated that HGF and WNT2 expressions increased accompanied by ID1's elevation (Figure 3B). In the *Id1* knockdown mouse, HGF and WNT2 shared the same expression pattern as ID1 did (Figure 4H). Considering the inhibition nature of ID1 on transcription, we speculated there might be an indirect approach by which ID1 upregulated WNT2 and HGF.

Recent researches emphasized the key role of CXCR7‐ID1 signaling in regeneration and proliferation in the liver. Activation of CXCR7‐ID1 signaling was enhanced by PH, and hepatic regeneration was elicited afterward.^51^ Accelerated liver regeneration was also observed in a BDL mouse model via enhanced CXCR7‐ID1‐WNT2/HGF signaling, while liver proliferation and mass regeneration were considerably decreased in *Cxcr7* deletion mice.^2^, ^51^ Acute‐on‐chronic liver failure (ACLF) is an acute deterioration of liver function in patients with cirrhosis.^52^ The liver of ACLF patients demonstrated a decrease in hepatocyte proliferation, along with the expression of CXCR7, ID1, and HGF.^53^ In the present study, *Id1* was upregulated in BDL and PH model. HGF and WNT2 were elevated and functioned antiapoptosis and proregeneration effects in PH model. In HepG2 cells, UDCA was shown the similar effects as it did in PH model. UDCA promoted the viability of HepG2 cells, and also increased *Id1*’s transcription and expression. These results gave us a hint that *Id1* upregulation was tightly correlated with UDCA treatment, along with the elevation of its downstream targets HGF and WNT2.

As discussed above, CXCR7‐ID1 signaling was important with regard to liver regeneration. On the other hand, numerous studies had reported that inhibition of CXCR4 results in antifibrotic effects. In patients with chronic hepatitis C, CXCR4 was increased in cirrhotic livers. Moreover, inhibition of CXCR4 resulted in decreased collagen I expression and stellate cell proliferation.^54^ CXCR4 expression was also negatively correlated to clinical outcome in patients with hepatocellular carcinoma.^55^ The CXCR4 antagonist AMD070 alleviated hepatic and pulmonary fibrosis in mice.^56^ Ding and other researchers had demonstrated that a conflict between the proregenerative CXCR7‐ID1 pathway and the profibrotic FGFR1‐CXCR4 pathway determined the direction toward either regeneration or fibrosis during chronic liver injury. ID1 was situated at the crossroads of these two pathways, deciding the result of this conflict.^2^, ^57^ Thus, ID1 was at the center of fibrosis versus regeneration regulation. CETSA is an assay by which the binding of drugs to their target proteins can be determined if obvious shifts are found in the melting curves of proteins. In other word, drugs can protect their (direct interact) target proteins from high temperature‐induced degradation.^28^ Similar technology, also known as the thermal shift assays, is also used for characterization of ligand binding in structural biology and drug screening.^58^, ^59^ In the present study, dramatic shift was found in the melting curves of ID1 protein incubated with UDCA, suggesting direct binding between them. A predicted model of the UDCA‐ID1 complex was also constructed (Figure 3E). However, more experiments are needed to verify direct interaction between UDCA and ID1.

With the use of a liver *Id1* knockdown mouse model, we demonstrated the role of ID1 in UDCA action. Our experiments indicated that *Id1* knockdown mice were more vulnerable to BDL surgery than control mice. More importantly, in *Id1* knockdown mouse, UDCA was unable to increase *Id1* expression as it did in BDL or PH model. Interfered ID1‐WNT2/HGF signaling thus reversed the therapeutical effects (antifibrosis and proregeneration) of UDCA. Collectively, these results provided a possible link between ID1, proregeneration, and antifibrosis. UDCA exerted antifibrosis effects in BDL mice, proregeneration effects in PH mice, and improved the thermal stability of ID1. However, UDCA failed to rescue fibrotic liver or to promote liver regeneration in liver *Id1* knockdown mice. Together, our findings indicated that UDCA provided protection against liver fibrosis through proregeneration via activation of the ID1‐WNT2/HGF signaling pathway.

## 5 CONCLUSIONIGS

Our current study reports a new UDCA mode of action, through which liver fibrosis is alleviated by proregeneration via activation of the ID1‐WNT2/HGF signaling pathway. Specifically, we affirmed the antifibrosis effects of UDCA in a BDL mice model. Next, we demonstrated that UDCA can enhance liver regeneration and CXCR7‐ID1‐WNT2/HGF signaling in a PH mouse model. UDCA protection against BDL was abolished in *Id1* knockdown mice, concomitant with impaired liver regeneration capacity, and decreased ID1‐WNT2/HGF signaling. Our results provide comprehensive evidence of the pharmacological effect of UDCA on liver fibrosis and regeneration. Overall, our results provide evidence that activation of ID1‐WNT2/HGF signaling could be the molecular mechanism underlying UDCA action.

## CONFLICTS OF INTEREST

The authors have declared no conflicts of interest.

## Supporting information

Supporting Information

Supporting Information

Supporting Information

Supporting Information

## Data Availability

Data available on request from the authors. The data that support the findings of this study are available from the corresponding author upon reasonable request.
