# Supplementary material for: Ursodesoxycholic acid alleviates liver fibrosis via proregeneration by activation of the ID1‐WNT2/HGF signaling pathway
Source: Clin Transl Med. 2021 Jan 24;11(2):e296. doi: 10.1002/ctm2.296 (PMC7828260; doi:10.1002/ctm2.296)
Supplement: Supplementary file 1 — Supporting Information [file CTM2-11-e296-s001.docx]

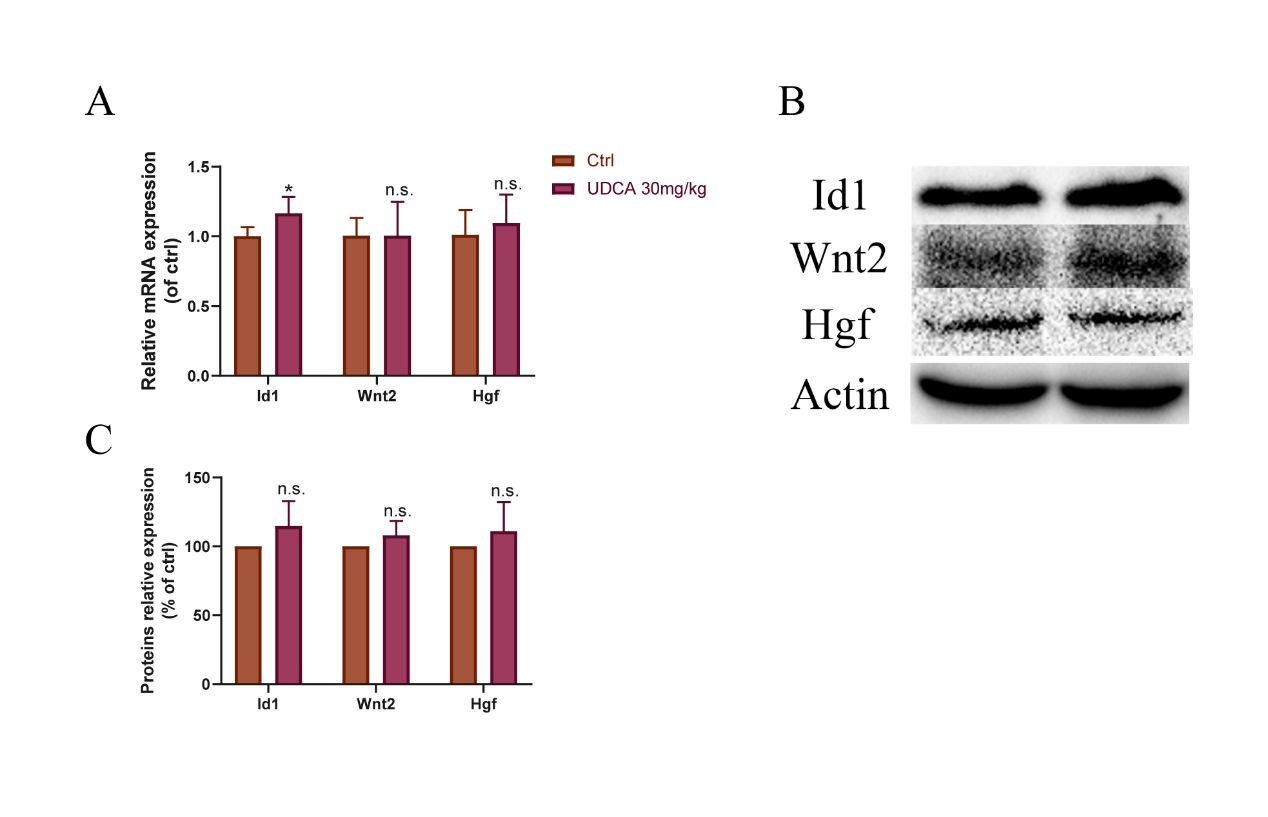


Figure S1. UDCA’s effects on ID1, WNT2, and HGF. Mice were treated with 30mg/kg UDCA for 7 days, and (A) *Id1*, *Wnt2*, and *Hgf*’s mRNA expression, as well as (B) ID1, WNT2, and HGF’s expressions were evaluated. (C) Statistical results of WB. All plots were shown as the mean ± SD. * *P* < 0.05, ** *P* < 0.01, *** *P* < 0.001, n.s., non-significant, comparing with the control group.


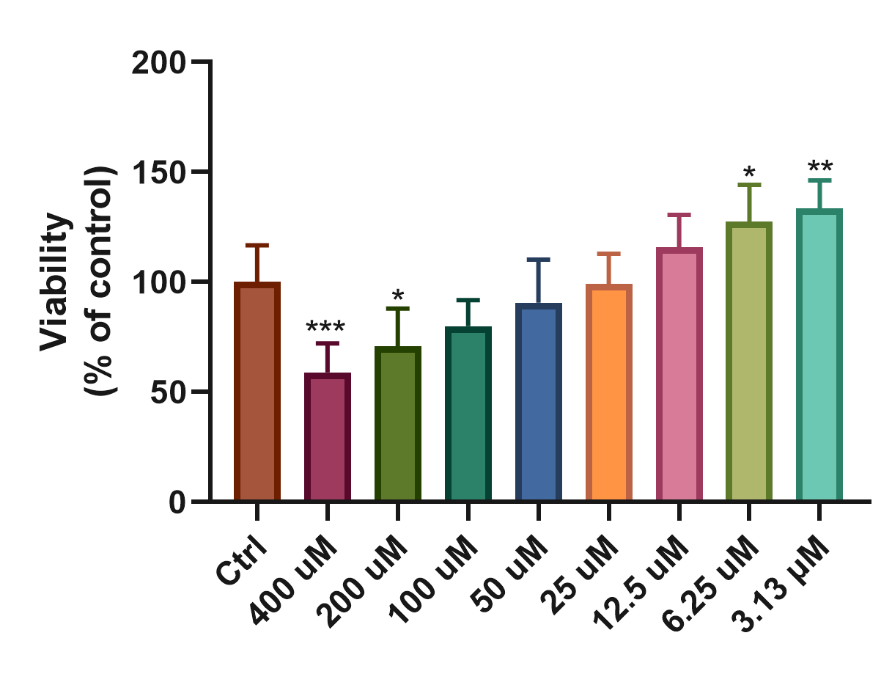


Figure S2. UDCA’s effects on HepG2 cells. All plots were shown as the mean ± SD. * P < 0.05, ** P < 0.01, *** P < 0.001, comparing with the control group.


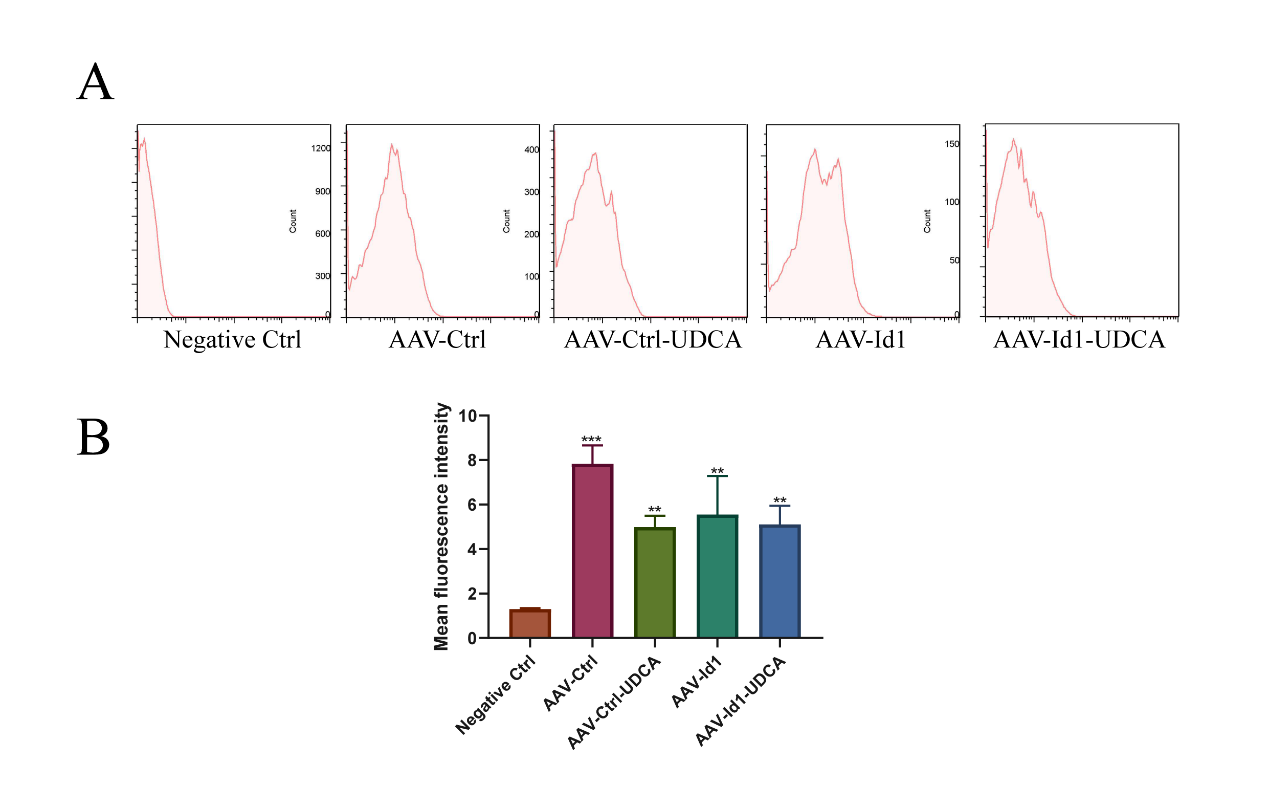


Figure S3. The verification of *Id1* knockdown. (A) Representative images of fluorescence analysis of liver cells. (B) Statistical result of fluorescence analysis. All plots were shown as the mean ± SD. * *P* < 0.05, ** *P* < 0.01, *** *P* < 0.001, comparing with the negative control group.
