# Supplementary figures and images for: Ursodesoxycholic acid alleviates liver fibrosis via proregeneration by activation of the ID1‐WNT2/HGF signaling pathway
Source: Clin Transl Med. 2021 Jan 24;11(2):e296. doi: 10.1002/ctm2.296 (PMC7828260; doi:10.1002/ctm2.296)

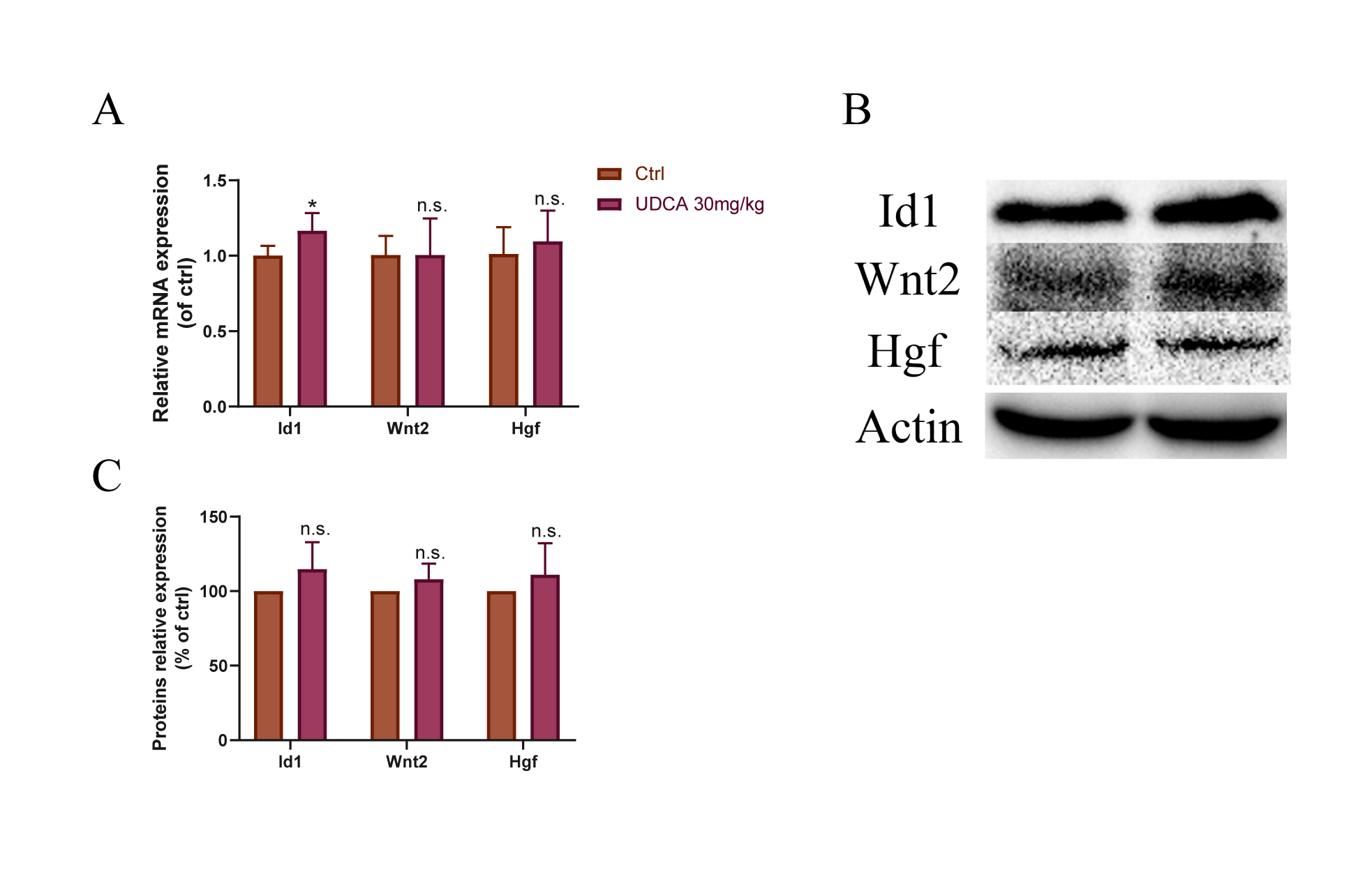

Supplement: Supplementary file 2 — Supporting Information [file CTM2-11-e296-s002.tif]

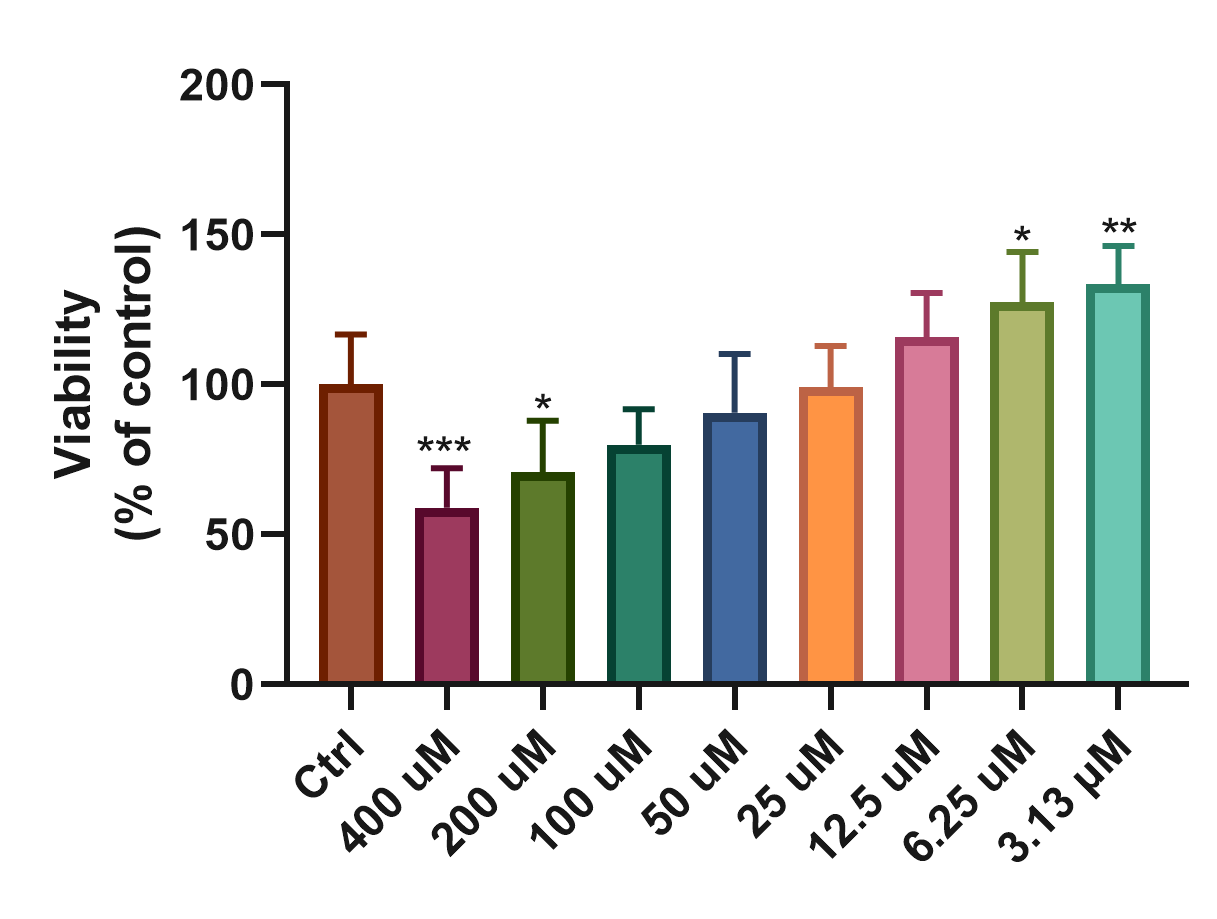

Supplement: Supplementary file 3 — Supporting Information [file CTM2-11-e296-s003.tif]

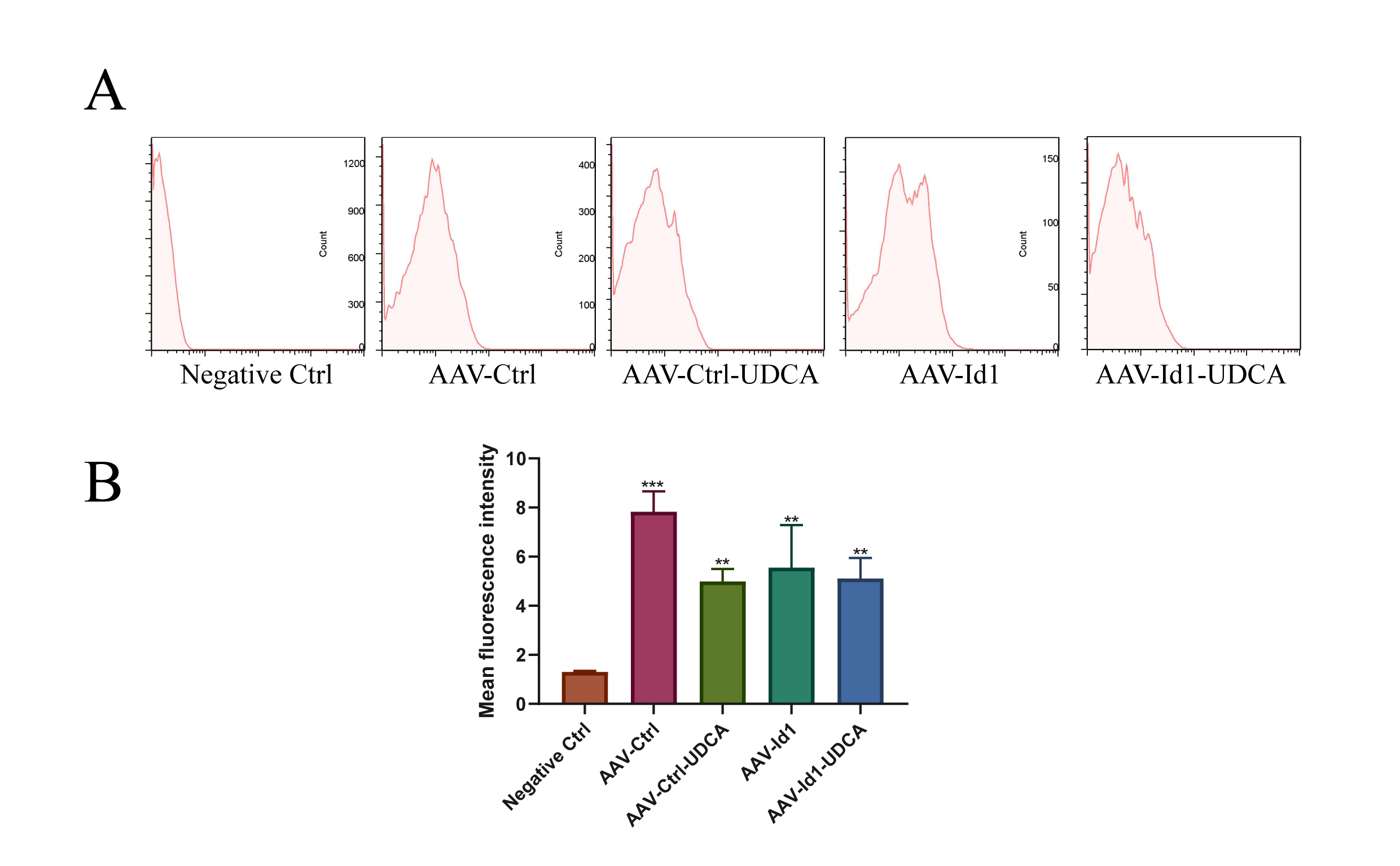

Supplement: Supplementary file 4 — Supporting Information [file CTM2-11-e296-s004.tif]
